# Effectiveness of Non-Adjuvanted Pandemic Influenza A Vaccines for Preventing Pandemic Influenza Acute Respiratory Illness Visits in 4 U.S. Communities

**DOI:** 10.1371/journal.pone.0023085

**Published:** 2011-08-12

**Authors:** Marie R. Griffin, Arnold S. Monto, Edward A. Belongia, John J. Treanor, Qingxia Chen, Jufu Chen, H. Keipp Talbot, Suzanne E. Ohmit, Laura A. Coleman, Gerry Lofthus, Joshua G. Petrie, Jennifer K. Meece, Caroline Breese Hall, John V. Williams, Paul Gargiullo, LaShondra Berman, David K. Shay, for the U.S. Flu-VE Network

**Affiliations:** 1 Department of Preventive Medicine, Vanderbilt University Medical Center, Nashville, Tennessee, United States of America; 2 Department of Medicine, Vanderbilt University Medical Center, Nashville, Tennessee, United States of America; 3 Department of Biostatistics, Vanderbilt University Medical Center, Nashville, Tennessee, United States of America; 4 Department of Pediatrics, Vanderbilt University Medical Center, Nashville, Tennessee, United States of America; 5 Department of Microbiology and Immunology, Vanderbilt University Medical Center, Nashville, Tennessee, United States of America; 6 Department of Epidemiology, School of Public Health, University of Michigan, Ann Arbor, Michigan, United States of America; 7 Marshfield Clinic Research Foundation, Marshfield, Wisconsin, United States of America; 8 Department of Medicine, University of Rochester School of Medicine and Dentistry, Rochester, New York, United States of America; 9 Department of Pediatrics, University of Rochester School of Medicine and Dentistry, Rochester, New York, United States of America; 10 Influenza Division, Centers for Disease Control and Prevention, Atlanta, Georgia, United States of America; University of Rochester, United States of America

## Abstract

We estimated the effectiveness of four monovalent pandemic influenza A (H1N1) vaccines (three unadjuvanted inactivated, one live attenuated) available in the U.S. during the pandemic. Patients with acute respiratory illness presenting to inpatient and outpatient facilities affiliated with four collaborating institutions were prospectively recruited, consented, and tested for influenza. Analyses were restricted to October 2009 through April 2010, when pandemic vaccine was available. Patients testing positive for pandemic influenza by real-time RT-PCR were cases; those testing negative were controls. Vaccine effectiveness was estimated in logistic regression models adjusted for study community, patient age, timing of illness, insurance status, enrollment site, and presence of high-risk medical conditions. Pandemic virus was detected in 1,011 (15%) of 6,757 enrolled patients. Fifteen (1%) of 1,011 influenza positive cases and 1,042 (18%) of 5,746 test-negative controls had record-verified pandemic vaccination >14 days prior to illness onset. Adjusted effectiveness (95% confidence interval) for pandemic vaccines combined was 56% (23%, 75%). Adjusted effectiveness for inactivated vaccines alone (79% of total) was 62% (25%, 81%) overall and 32% (−92%, 76%), 89% (15%, 99%), and −6% (−231%, 66%) in those aged 0.5 to 9, 10 to 49, and 50+ years, respectively. Effectiveness for the live attenuated vaccine in those aged 2 to 49 years was only demonstrated if vaccination >7 rather than >14 days prior to illness onset was considered (61%∶ 12%, 82%). Inactivated non-adjuvanted pandemic vaccines offered significant protection against confirmed pandemic influenza-associated medical care visits in young adults.

## Introduction

In April 2009, human infections with a novel influenza A (H1N1) virus were first detected in the US [1], with declaration of a global pandemic by June 2009. By February 2010, 30 different monovalent pandemic vaccines were licensed world-wide [2], including four distributed in the US, which were analogous to previously licensed seasonal influenza vaccines. The US inactivated vaccines licensed were non-adjuvanted and differed from those licensed in Europe and Canada, where adjuvanted vaccines predominated. A single dose of the inactivated US vaccines generally elicited antibody responses associated with protection in vaccinees aged 10 years and older; however, a second dose was required to achieve seroprotection among a high proportion of young children [2], [3].

Pandemic vaccines became available in the US by October 2009. Target groups for limited initial doses included health care workers, pregnant women, close contacts of children aged <6 months, all persons aged 6 months to 24 years, and persons 25 through 64 years with high-risk medical conditions [4]. By mid to late December, vaccine was available for all, and about 125 million doses were eventually distributed.

We assessed the effectiveness of pandemic vaccines against laboratory confirmed pandemic influenza associated health care visits in four US communities through the Centers for Disease Control and Prevention's (CDC) Influenza Vaccine Effectiveness (Flu-VE) Network.

## Methods

### Subject enrollment

We enrolled persons seeking care for acute respiratory illness at medical facilities affiliated with the Marshfield Clinic and St. Joseph's Hospital, Marshfield, WI; the University of Michigan Health System, Ann Arbor, MI and Henry Ford Health System, Detroit, MI; the University of Rochester (Strong Memorial and Rochester General Hospitals), NY; and Vanderbilt University, Summit, St. Thomas and Baptist Hospitals, Nashville, TN. Patients were prospectively enrolled from 9/1/2009 through 5/31/2010. Analyses included subjects aged ≥6 months who were enrolled at least 7 days after the first pandemic H1N1 vaccination in an enrolled patient through 7 days following the last pandemic influenza diagnosis in each study site: 10/06/09–12/21/09 (NY), 10/08/09–3/19/10 (WI), 10/09/09–4/9/10 (TN), and 10/23/09–4/26/10 (MI).

The source populations included community-dwelling residents in Marshfield, seen in primary care clinics or hospitals affiliated with Marshfield Clinic; patients receiving care at selected University of Michigan Health System or Henry Ford Health System outpatient clinics or their affiliated hospitals; residents of Monroe County (Rochester), NY admitted to two area hospitals or emergency departments, or seen at 1 adult and 3 children's outpatient clinics; and residents of Davidson (Nashville) and surrounding counties admitted to four area hospitals, two associated emergency departments, or seen at 1 adult and 1 children's outpatient clinics. Patients presenting with acute respiratory illness were recruited by trained personnel 2–6 days per week, depending on location and staffing, and were identified by screening procedures (review of electronic records or other lists of admission diagnoses or symptoms).

Potentially eligible patients (or parents/guardians) were approached by trained staff to assess eligibility and obtain informed consent. Each consented participant (or parent/guardian) completed an interview to ascertain symptoms and date of symptom onset. Age, sex, self-reported race, insurance status, and history of chronic medical conditions were ascertained from interview and/or medical record review. Persons were defined as high risk if they had documented medical conditions that increase the risk of influenza complications, as defined by the Advisory Committee on Immunization Practices [5]. Weight and height were not uniformly collected; thus, obesity data were not available.

In three communities, receipt of pandemic and seasonal 2009–10 influenza vaccines was ascertained by patient or parental report and confirmed by medical record review and/or State vaccine registries. In Wisconsin, vaccine receipt was confirmed by a vaccine registry which captures 95% of all influenza (including pandemic) vaccinations in that population [6], [7].

Study procedures, informed consent documents and data collection forms were reviewed and approved by Institutional Review Boards of Marshfield Clinic, University of Michigan, University of Rochester, and Vanderbilt University, St Thomas and Baptist Hospitals (Sterling), and Summit Hospital (Western), and the Centers for Disease Control and Protection. Written consent/assent was obtained from all study participants and/or their parents/guardians.

### Laboratory methods

Respiratory specimen swabs collected from each enrolled patient were tested at the study sites using CDC's real-time RT-PCR (rRT-PCR) protocol for detection and characterization of influenza viruses using dual-labeled probe (Taqman®) chemistry. CDC provided primers, probes, and control materials, and a proficiency testing panel was completed by each site. A sample of rRT-PCR-positive specimens was cultured using MDCK cells and a subset of viral isolates was antigenically characterized by CDC using a hemagglutination inhibition assay with a panel of standard reference viruses and the corresponding post-infection ferret antisera [8].

### Vaccine effectiveness estimates

The primary outcome was medically attended acute respiratory illness with rRT-PCR confirmed pandemic influenza virus detected. The primary exposure was receipt of a single pandemic vaccine >14 days before illness onset. Vaccine effectiveness was estimated as 100%×(1−adjusted odds ratio) using logistic regression models. The primary model included all pre-specified potential confounders. Day of symptom onset was modeled as days since pandemic vaccine availability using linear tail-restricted cubic spline functions, with four knots at 5%, 35%, 65% and 95% quantiles. Spline functions can describe continuous data well, while economizing on the degrees of freedom used [9]. Other pre-specified potential confounders included: age, study community, insurance status, enrollment site, and presence of high-risk medical conditions. Stratified analyses were performed by age category: 6 months to <10 years, because one dose of vaccine was considered partial vaccination for this group; 10–49 years, as a single dose resulted in high levels of seroprotection in this group [2], [3]; and 50 years and older, a group with relatively low pandemic influenza attack rates. We also examined effectiveness of inactivated pandemic and live attenuated vaccines separately. We did not perform analyses by study community or enrollment site because of limited sample size. Secondary analyses used a 7- rather than 14-day period between vaccination and illness onset to define immunization, since seroprotective levels are often evident within the first week following vaccination [10], [11]. We also evaluated the effectiveness of the 2009–2010 seasonal vaccines for prevention of pandemic illness, controlling for receipt of pandemic vaccine. Finally, because of strong confounding by age and illness onset date relative to vaccine availability, an alternate analytic model was developed, which adjusted for age and time using categories, with linear adjustment within categories rather than spline functions (supplementary Table S1).

Analyses were conducted using R 2.10.1 (r-project.org) for descriptive statistics and the primary model and SAS 9.1 software (SAS Institute, Cary, NC) for the alternate analytic model. A 95% confidence interval (CI) was calculated for each estimate; if this interval excluded 0%, the estimate was considered statistically significant. Data are expressed as median (interquartile range [IQR]) or frequency (percentage). Comparisons between cases and controls, and between vaccinated and unvaccinated patients used Wilcoxon rank-sum test for continuous variables and chi-square test for categorical variables.

## Results

Of 10,004 patients with medically attended acute respiratory illness enrolled from September 2009 through May 2010, 6,757 (68%) patients were included in case-control analyses. Exclusions were for inconclusive laboratory results (n = 33), age <6 months (n = 321), enrollment before pandemic vaccine availability (n = 1320) or following the last pandemic influenza diagnosis (n = 1442), and non-verified vaccination status (n = 131).

**Figure 1:**
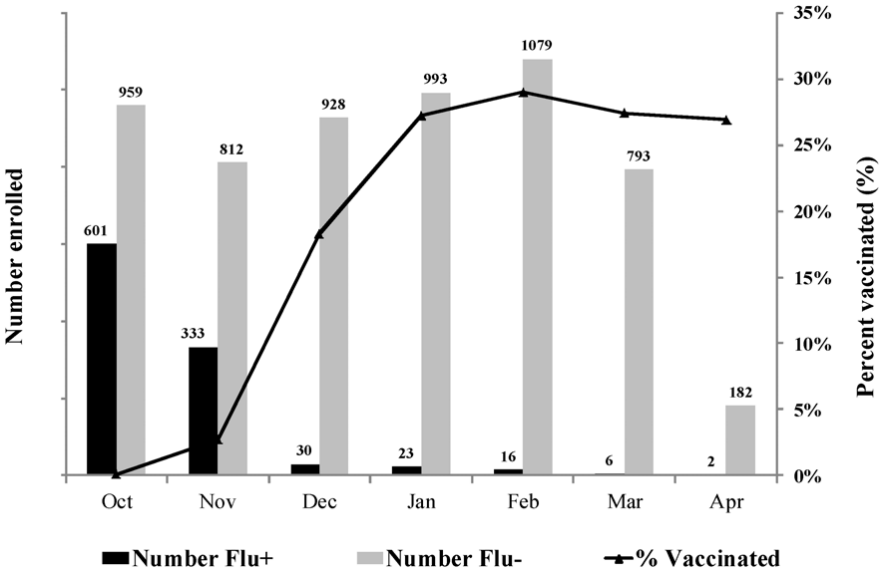
Number of influenza-positive cases and influenza-negative controls enrolled in the Flu-VE study by month of illness onset, October 2009 through April 2010, and percent vaccinated greater >14 days prior to illness onset. (Vaccination rates were <1% in October).

Of 6,757 study patients, 1,011 (15%) tested positive for pandemic influenza virus. CDC further characterized all 70 cultured and submitted viruses as A/California/07/2009-like (H1N1). Pandemic virus circulation peaked at study sites just as vaccine became available. During October, <1% and 3% of enrolled controls had been vaccinated >14 and >7 days before illness onset, respectively. Vaccination levels rose quickly and approached 27% in controls overall, remaining stable from January through April 2010 (Figure 1).

The distribution of cases and controls differed by study community (Table 1) in part due to timing of the pandemic, with the last pandemic virus detected in December 2009 in Rochester compared with March or April 2010 for the other 3 communities. In addition to the strong association between case status and calendar time (Figure 1), influenza positive and negative patients differed in other ways (Table 1). Compared with controls, cases were younger (median age 13 vs. 29 years), and less likely to be Black (14% vs. 20%), to have a high-risk medical condition (32% vs. 46%), or to be vaccinated. Cases were more likely to have private insurance (71% vs. 60%) and be enrolled at an outpatient setting (79% vs. 58%). Influenza positive cases also had shorter symptom duration at presentation (63% vs. 44% with symptoms <3 days).

**Table 1:** Descriptive Characteristics of Enrolled Patients with Medically Attended Acute Respiratory Illness by Case Control Status.

Characteristics	Influenza Positive Cases		Influenza Negative Controls		
	N = 1,011	%	N = 5,746	%	P Value
**Study community**					<0.001^1^
**Marshfield, WI**	533	53%	2,388	42%	
**Rochester, NY**	178	18%	259	5%	
**Southeast, MI**	198	20%	1,086	19%	
**Nashville, TN**	102	10%	2,013	35%	
**Age group**					<0.001^1^
**6 months–9 years**	377	37%	1,791	31%	
**10–49 years**	536	53%	2,193	38%	
**50+ years**	98	10%	1,762	31%	
**Median (IQR)**	6 **13** 28		6 **29** 54		<0.001^2^
**Sex**					0.086^1^
**Female**	536	53%	3,216	56%	
**Male**	475	47%	2,532	44%	
**Race** 3					<0.001^1^
**White**	702	74%	4133	73%	
**Black**	133	14%	1099	20%	
**Other**	119	12%	394	7%	
**Insurance status**					<0.001^1^
**None**	83	8%	290	5%	
**Private**	720	71%	3,427	60%	
**Public only**	208	21%	2,029	35%	
**Enrollment site**					<0.001^1^
**Outpatient Clinic**	802	79%	3,326	58%	
**Emergency Room**	117	12%	739	13%	
**Inpatient**	92	9%	1,681	29%	
**High-risk condition**					<0.001^1^
**No**	692	68%	3,126	54%	
**Yes**	319	32%	2,620	46%	
**Onset to test**					<0.001^1^
**<3 days**	632	63%	2,516	44%	
**3–6 days**	338	33%	2,442	42%	
**7+ days**	41	4%	788	14%	
**Vaccine (interval)**					
**Pandemic (14 days)**	15	1%	1042	18%	<0.001^1^
**Pandemic (7 days)**	22	2%	1107	19%	<0.001^1^
**Seasonal (14 days)**	203	20%	2182	38%	<0.001^1^

1 Pearson test;

2 Wilcoxon test;

3 missing data for race (n = 177).

Of the 1057 vaccinated patients, 21% received live attenuated vaccine and 79% received one of three inactivated vaccines, including sanofi-pasteur (48%), Novartis (22%), CSL (6%), and manufacturer unknown (4%). In addition to the strong association between vaccination and time (Figure 1), vaccinated patients were younger than unvaccinated patients (median 11 vs. 27 years); less likely to be Black (10% vs. 20%); and more likely to be insured, enrolled in outpatient settings, and to have a longer symptom duration prior to presentation (42% vs. 47% with symptoms <3 days) (Table 2). Few vaccinees tested positive for pandemic influenza (1% vs. 17% for unvaccinated patients).

**Table 2:** Descriptive Characteristics of Enrolled Patients with Medically Attended Acute Respiratory Illness by Pandemic Vaccine Status.

Characteristics	Vaccinated		Unvaccinated		
	N = 1,057	%	N = 5,700	%	P Value
**Study community**					<0.001^1^
**Marshfield, WI**	578	55%	2,343	41%	
**Rochester, NY**	15	1%	422	7%	
**Southeast, MI**	166	16%	1,118	20%	
**Nashville, TN**	298	28%	1,817	32%	
**Age group**					<0.001^1^
**6 months–9 years**	513	49%	1,655	29%	
**10–49 years**	297	28%	2,433	43%	
**50+ years**	247	23%	1,613	28%	
**Median (IQR)**	2 **11** 47		7 **27** 52		<0.001^2^
**Sex**					0.996^1^
**Female**	587	56%	3,165	56%	
**Male**	470	44%	2,535	44%	
**Race** 3					<0.001^1^
**White**	829	79%	4006	72%	
**Black**	106	10%	1126	20%	
**Other**	109	10%	404	7%	
**Insurance status**					<0.001^1^
**None**	25	2%	348	6%	
**Private**	677	64%	3,470	61%	
**Public only**	355	34%	1,882	33%	
**Enrollment site**					<0.001^1^
**Outpatient**	739	70%	3,389	59%	
**Emergency Dept**	82	8%	774	14%	
**Inpatient**	236	22%	1,537	27%	
**High-risk condition**					0.644^1^
**No**	604	57%	3,214	56%	
**Yes**	453	43%	2,486	44%	
**Symptom duration**					<0.001^1^
**<3 days**	444	42%	2,704	47%	
**3–6 days**	475	45%	2,305	40%	
**7+ days**	138	13%	691	12%	
**Influenza test**					<0.001^1^
**Positive**	15	1%	996	17%	
**Negative**	1042	99%	4704	83%	

1 Pearson test;

2 Wilcoxon test;

3 missing data for race (n = 177).

Overall vaccine effectiveness was 56% (95% CI, 23%–75%) adjusting for study community, date of illness onset, age, insurance status, enrollment site, and presence of high-risk medical conditions (Table 3). Controlling for race (missing in 177 patients) yielded identical results. The crude vaccine effectiveness estimate of 93% (based on the proportion of vaccinated case and controls) was biased because pandemic influenza cases were skewed to the earlier time when overall vaccination levels were low (Figure 1); adjusting for calendar time was essential for all analyses. Given the strong temporal trends with low numbers of cases during the period of greatest vaccine availability, power for any stratified analyses was limited. None of the age-specific adjusted analyses indicated significant vaccine effectiveness. Too few children aged <10 years received two doses of vaccine to estimate effectiveness separately for this group.

**Table 3:** Percent Vaccinated more than 14 Days Prior to Illness Onset by Case Control Status and Adjusted Vaccine Effectiveness, by Age Group and Vaccine Type.

Age (Years)	Influenza Positive Cases %Vaccinated	Influenza Negative Controls (N Vaccinated/Total)	%Adjusted Vaccine Effectiveness^1^ (95% Confidence Interval)
	**a. Any Pandemic Vaccine**		
**All**	1.5 (15/1011)	18.1 (1042/5746)	55.9 (22.7, 74.8)
**0.5–9**	1.6 (6/377)	28.3 (507/1791)	40.6 (−51.7, 76.7)
**10–49**	0.9 (5/536)	13.2 (292/2193)	60.9 (−0.5, 84.8)
**≥50**	4.1 (4/98)	13.7 (243/1762)	−5.8 (−230.5, 66.1)
	**b. Inactivated Pandemic Vaccine** 2		
**All**	1.0 (10/999)	15.0 (826/5504)	61.7 (24.9, 80.5)
**0.5–9**	1.3 (5/373)	22.8 (376/1647)	31.6 (−91.9, 75.6)
**10–49**	0. 2 (1/528)	9.9 (208/2096)	88.6 (15.2, 98.5)
**≥50**	4.1 (4/98)	13.7 (242/1761)	−5.9 (−230.9, 66.1)
	**c. Live Attenuated Pandemic Vaccine** 3		
**2–49**	0.6 (5/860)	7.3 (214/2931)	39.9 (−56.4, 76.9)
**2–9**	0.3 (1/330)	13.3 (130/977)	54.8 (−269.5, 94.5)
**10–49**	0.8 (4/530)	4.3 (84/1954)	−1.0 (−196.5, 65.6)

1 Adjusted for study community, cubic spline of age and time since 10/04/09 in days, insurance status, enrollment site, presence of high risk condition;

2 Excludes all with live attenuated vaccination prior to illness onset;

3 Excludes all with inactivated pandemic vaccination prior to illness onset and those not aged 2 to 49 years.

Overall results were similar when a 7-day interval defined immunization (Table 4). Significant vaccine effectiveness was demonstrated overall (58%, 95% CI, 32%–74%) and for those aged 10 to 49 years (59%, 95% CI, 15%–80%). Results using an alternate model to control for age and for timing of illness yielded similar results (supplemental Table S1). In contrast to pandemic vaccine, receipt of seasonal vaccine did not reduce medically attended visits associated with pandemic virus infection (vaccine effectiveness 11% (95% CI −9%–27%) (supplemental Table S1).

**Table 4:** Percent Vaccinated more than 7 Days Prior to Illness Onset by Case Control Status and Adjusted Vaccine Effectiveness, by Age Group and Vaccine Type.

Age (Years)	Influenza Positive Cases %Vaccinated	Influenza Negative Controls (N Vaccinated/Total)	%Adjusted Vaccine Effectiveness^1^ (95% Confidence Interval)
	**a. Any Pandemic Vaccine**		
**All**	12.2 (22/1011)	19.3 (1107/5746)	57.9 (32.4, 73.7)
**0.5–9**	2.4 (9/377)	29.6 (531/1791)	50.8 (−8.4, 77.7)
**10–49**	1.8 (9/536)	14.2 (312/2193)	59.0 (14.7, 80.3)
**≥50**	4.1 (4/98)	15.0 (264/1762)	22.3 (−134.8, 74.3)
	**b. Inactivated Pandemic Vaccine** 2		
**All**	1.0 (14/999)	15.9 (875/5504)	58.6 (26.2, 76.7)
**0.5–9**	1.3 (7/373)	23.7 (390/1647)	15.9 (−107.7, 66.0)
**10–49**	0. 2 (3/528)	10.6 (222/2096)	77.2 (24.8, 93.1)
**≥50**	4.1 (4/98)	14.9 (263/1761)	22.2 (−135, 74.3)
	**c. Live Attenuated Pandemic Vaccine** 3		
**2–49**	0.9 (8/860)	7.8 (230/2931)	60.6 (12.3, 82.3)
**2–9**	0.6 (2/330)	14.3 (140/977)	81.9 (13.6, 96.2)
**10–49**	1.1 (6/530)	4.6 (90/1954)	26.4 (−91.3, 71.7)

1 Adjusted for study community, cubic spline of age and time since 10/04/09 in days, insurance status, enrollment site, presence of high risk condition;

2 Excludes all with live attenuated vaccination prior to illness onset;

3 Excludes all with inactivated pandemic vaccination prior to illness onset and those not aged 2 to 49 years old.

Adjusted effectiveness of inactivated vaccines (79% of total) was 62% (95% CI, 25%–81%) overall and 89% (95% CI, 15%–99%) among those aged 10 to 49 years. Using a 7- rather than 14-day interval, estimates were modestly lower but significant overall (59%, 95% CI, 26%–77%) and for those aged 10 to 49 years (77%, 95% CI, 25%–93%).

Live attenuated vaccine was evaluated among those aged 2 to 49 years, for whom the vaccine is licensed. Significant effectiveness was not demonstrated in the primary analysis. Using a 7-day interval, vaccine effectiveness was estimated as 61% (95% CI, 12%–82%) among those aged 2–49 years and 82% (95% CI, 14%–96%) among those aged 2 to 9 years (Tables 3 and 4).

## Discussion

We prospectively identified >1000 patients with pandemic influenza-associated illness resulting in a medical encounter following availability of US pandemic vaccines. Pandemic vaccines offered significant overall protection against medically attended influenza illnesses. These findings in four geographically and economically diverse communities are relevant to the entire US. The timing of pandemic virus-associated illness in the combined study communities reflected that of the rest of the US, with a first peak in June–July 2009, prior to vaccine availability, and a second peak in September-November 2009, with low virus circulation during the remainder of the winter [12]. Vaccination patterns in study sites also mirrored national trends. CDC estimated state-specific median vaccine coverage as of January 31, 2010 to be 23.9% (range 12.9% to 38.8%), consistent with our observations [9].

One dose of pandemic vaccine was associated with an overall effectiveness of 56%. For inactivated vaccines, estimated effectiveness was 89% (95% CI, 15%–99%) among those aged 10–49 years, but significant effectiveness was not demonstrated in other age groups. Relatively good vaccine effectiveness would be expected in healthy young adults for the pandemic vaccine that was well matched to the circulating strain. However, the wide confidence interval suggests caution with interpretation of our point estimate. Results from clinical trials conducted during several recent seasons indicated that inactivated vaccines performed better than live attenuated vaccines among healthy young adults [13]–[15]. Lack of effectiveness of one dose of inactivated vaccines in young children is not surprising, since two doses are recommended and were often necessary for seroconversion [2], [3].

We had insufficient power to adequately assess live attenuated vaccine in adults or to compare vaccines. Use of a 7-day window increased the number of vaccinated persons, increasing study power. One dose of live attenuated pandemic vaccine was estimated to be 82% effective in children aged 2 to 9 years, but no effectiveness was demonstrated in those 10 to 49 in any analysis. This finding also meets expectations, since live attenuated vaccines have had high effectiveness in young children following both one and two doses [16].

In contrast to Europe and Canada, the US opted not to use adjuvanted vaccines for pandemic response. Such vaccines had never been licensed in the United States and their use for emergency response could have further delayed vaccination efforts. In addition, immunogenicity of non-adjuvanted inactivated vaccines was demonstrated in clinical trials [2], [3]. Evaluation of non-adjuvanted vaccines is important, since they may have fewer local and systemic reactions, but can be less immunogenic in some populations [17]. One dose of inactivated pandemic vaccine resulted in hemagglutination inhibition (HI) titers ≥40 by 21 days in >90% of study subjects aged 10 years or older; whereas two doses were required to achieve this level in younger children [2], [18]–[21].

The only other large study of unadjuvanted vaccine was conducted in Chinese school children, 78% of who were aged 12–17 years. The reported effectiveness was 87% (95% CI, 75%–93%). Methodologic concerns have been raised suggesting that this study may have overestimated of effectiveness [22], [23]. Nonetheless, the results are consistent with our results for inactivated US vaccine among those aged 10–49 years. Other studies have evaluated primarily adjuvanted pandemic vaccines. German and Spanish studies reported very high effectiveness; however, neither accounted for differences between vaccinated and unvaccinated persons [24], [25]. A small controlled Canadian study suggested that one dose of adjuvanted vaccine was effective for young children [26]. A study of 933 pandemic influenza cases and 1220 test negative controls in England found overall effectiveness of 62% (95% CI 33%–78%) for adjuvanted vaccine, with significant effectiveness in children <10 years (77%, 95% CI 11%–94%) and those aged 10–24 years (100%, 80%–100%) [27]. Finally, a multicenter study based on sentinel practitioner surveillance (918 cases and 1984 test-negative controls) in seven European countries (80% adjuvanted vaccine) reported an adjusted vaccine effectiveness of 72% (95% CI 46%–86%) [28].

Our study was limited by the few influenza illnesses after pandemic vaccines became available, which diminished the power overall and for subgroup analyses. Nonetheless, the consistency of the findings in all statistical models, the finding of the most robust effect in those 10 to 49 years with inactivated vaccines, the effectiveness of live attenuated vaccine in young children in secondary analyses, and the lack of effectiveness of seasonal influenza vaccines, suggests that pandemic vaccines licensed for use in the United States provided significant protection against medically attended pandemic influenza illness.

Observational studies complement data from clinical trial data to provide a more complete picture of influenza vaccine effectiveness. Clinical trials for licensure of inactivated vaccines rely in part on immunogenicity data which are not ideal predictors of efficacy. Lack of randomization in observational studies necessitates examination and control for factors that differ systematically by both vaccination and case status. In our population, age and timing of illness were the only factors strongly associated with both vaccination and pandemic virus associated illness. These as well as study community were accounted for in both the primary and alternate models. Use of the test-positive case vs. test-negative control methodology has the additional advantage of controlling for difficult to measure factors associated with both illness severity and the propensity to seek care when ill [29], [30].

Our results suggest that a single dose of a US licensed non-adjuvanted pandemic vaccine was capable of preventing over half of medical care visits associated with pandemic virus infection, and that inactivated vaccines were very effective for those aged 10 to 49 years. Few children aged <10 years received 2 doses, which limited effectiveness in this age group, and likely decreased the overall effectiveness estimate, given the high pandemic attack rate among young children. Despite significant effectiveness, overall vaccine impact was limited by timing of its availability.

Consistently conducted annual vaccine effectiveness will be needed to assess the clinical effectiveness of new seasonal influenza vaccines, which may be licensed for use in the US based on enhanced immunogenicity alone (http://www.fda.gov/NewsEvents/Newsroom/PressAnnouncements/ucm195483.htm). In order to make vaccine- and age-specific estimates of vaccine effectiveness, large annual studies should be a priority, given the universal US recommendation for influenza vaccination.

## Supporting Information

Table S1 Percent Vaccinated more than 14 Days Prior to Illness Onset by Case Control Status and Adjusted Vaccine Effectiveness (alternate model), by Age Group and Vaccine Type.
(DOC)
