# Supplementary material for: Effectiveness of Non-Adjuvanted Pandemic Influenza A Vaccines for Preventing Pandemic Influenza Acute Respiratory Illness Visits in 4 U.S. Communities
Source: PLoS One. 2011 Aug 12;6(8):e23085. doi: 10.1371/journal.pone.0023085 (PMC3155536; doi:10.1371/journal.pone.0023085)
Supplement: Table S1 — Percent Vaccinated more than 14 Days Prior to Illness Onset by Case Control Status and Adjusted Vaccine Effectiveness (alternate model), by Age Group and Vaccine Type. (DOC) [file pone.0023085.s001.doc]

|  | | | | | | |
| --- | --- | --- | --- | --- | --- | --- |
| **Table S. Percent Vaccinated more than 14 Days Prior to Illness Onset by Case Control Status and Adjusted Vaccine Effectiveness (alternate model), by Age Group and Vaccine Type.** | | | | | | |
| **Age (Years)** | | **Influenza Positive Cases %Vaccinated** | | **Influenza Negative Controls (N Vaccinated/Total)** | **%Adjusted Vaccine Effectiveness^1^ (95% Confidence Interval)** | |
|  | 1. **Any Pandemic Vaccine^1^** | | | | |  |
| **All** | 12.2 (22/1011) | | 19.3 (1107/5746) | | 49.3 (10.6, 71.3) | |
| **0.5 —9** | 2.4 (9/377) | | 29.6 (531/1791) | | 26.0 (-95.1, 71.9) | |
| **10 —49** | 1.8 (9/536) | | 14.2 (312/2193) | | 63.0 (4.7, 85.6) | |
| **>50** | 4.1 (4/98) | | 15.0 (264/1762) | | -13.8 (-262.7, 64.3) | |
|  | 1. **Any Seasonal Vaccine^2^** | | | | | |
| **All** | 20.1 (203/1012) | | 40.0 (2182/5746) | | | 11.0 (-8.8, 27.3) |
| **0.5—9** | 24.9 (94/377) | | 43.9 (787/1791) | | | 4.4 (-34.1, 31.9) |
| **10 —49** | 15.1 (81/537) | | 26.4 (578/2193) | | | 9.3 (-24.1, 33.7) |
| **>50** | 28.6 (28/98) | | 46.4 (817/1762) | | | 8.1 (-55.1, 45.6) |
| ^1^Adjusted for study community, linear piecewise function for age group 6 months to 4 years, 5 to 8, 9 to 18, 19 to 29, 30 to 39, 40 to 49, 50 to 64, and 65 or more years, age and age group interaction, and onset date in 2 week intervals since 10/04/09, except February 1 to April 30, which was considered one interval; ^2^ Adjusted for study community, cubic spline of age and time since 10/04/09 in days, insurance status, enrollment site, presence of high risk condition. | | | | | | |
